# Functional Gut Microbiota Alterations in Patients with Inflammatory Bowel Disease Receiving Long-Term Biologic Therapy: A Pilot Comparative Study

**DOI:** 10.3390/jcm15187270

**Published:** 2026-09-18

**Authors:** Sergiu Ioan Frandeș, Oana Frandeș, Melania Macarie, Simona Maria Bățagă

**Affiliations:** 1 Doctoral School of Medicine and Pharmacy, George Emil Palade University of Medicine, Pharmacy, Science, and Technology of Targu Mures, 540142 Targu Mures, Romania; sergiu.frandes@umfst.ro (S.I.F.); oana.frandes@umfst.ro (O.F.); 2 Department of Internal Medicine I, George Emil Palade University of Medicine, Pharmacy, Science, and Technology of Targu Mures, 540142 Targu Mures, Romania; simona.bataga@umfst.ro

**Keywords:** inflammatory bowel disease, biologic therapy, functional microbiome, microbial dysbiosis, short-chain fatty acids, intestinal barrier, 16S rRNA sequencing

## Abstract

**Background:** Biological therapies have substantially improved clinical outcomes in inflammatory bowel disease (IBD); however, the relationship between long-term biologic therapy, disease remission, and gut microbiome characteristics remains incompletely understood. This pilot study aimed to characterize the taxonomic composition and taxonomically inferred functional microbiome features of patients with IBD receiving long-term biologic therapy and achieving clinical and biological remission at the time of microbiome assessment. **Methods:** Patients with IBD receiving long-term biologic therapy and achieving clinical and biological remission at the time of microbiome assessment were prospectively enrolled. Gut microbiome composition was assessed using 16S rRNA gene sequencing. Functional microbiome profiling included microbial diversity (Shannon index), fecal pH, Firmicutes/Bacteroidetes ratio, and bacterial groups associated with short-chain fatty acid production, mucin degradation, and lipopolysaccharide (LPS) production. Comparative analyses between disease phenotypes and biologic therapies were performed using non-parametric statistical methods. **Results:** Fifteen patients with IBD were included, comprising eight patients with ulcerative colitis and seven with Crohn’s disease. Several microbiome features were outside the laboratory-specific reference intervals despite clinical and biological remission at the time of microbiome assessment. Shannon diversity values were below the laboratory-specific reference threshold in 60.0% of patients, while taxa associated with mucin degradation, lactate production, and LPS production were outside the respective reference intervals in 66.7%, 53.3%, and 46.7% of patients. The *Firmicutes*/*Bacteroidetes* ratio was outside the reference interval in 46.7% of patients. The gut microbiota was predominantly composed of *Firmicutes* and *Bacteroidetes*, with considerable interindividual variability in taxonomic composition and taxonomically inferred functional features. No statistically significant differences were identified between Crohn’s disease and ulcerative colitis or between biologic therapies after false discovery rate correction. **Conclusions:** Patients with IBD receiving long-term biologic therapy and achieving clinical and biological remission at the time of microbiome assessment exhibited substantial interindividual variability in taxonomic composition and taxonomically inferred functional microbiome features. Several parameters were outside laboratory-specific reference intervals; however, the cross-sectional design and absence of pre-treatment and matched healthy control samples preclude conclusions regarding microbiome restoration or treatment-related effects. These findings support further longitudinal investigation of microbiome characteristics during biologic therapy.

## 1. Introduction

Crohn’s disease and ulcerative colitis are chronic, recurrent inflammatory diseases that affect the gastrointestinal tract and are encountered with increased frequency, especially in Europe (ulcerative colitis with a prevalence of 0.505% and Crohn’s disease with a prevalence of 0.322%) [1,2,3]. Among the most studied etiologies implicated in the development of inflammatory bowel diseases are environmental factors, immune factors, genetic susceptibility, and intestinal microbiota [4].

Regarding inflammatory bowel diseases (IBDs), the microbiome or microbiota is a complex group of Archaea, bacteria, fungi, viruses, and helminths that live in the body and also influence the symptoms, flare-ups, remission, and response to treatment of IBD [5].

Numerous studies have demonstrated and confirmed the existence of a different composition of the intestinal microbiota in patients with IBD compared to healthy patients [6]. The main changes observed in the microbiota composition of these patients are a reduction in commensal bacteria with anti-inflammatory roles and an increase in species with pro-inflammatory potential. The depletion of several butyrate-producing and SCFA-associated bacteria, including *Faecalibacterium prausnitzii*, *Roseburia intestinalis*, *Eubacterium hallii, Gemmiger formicilis*, *Eubacterium rectale*, and *Ruminococcus bromii*, has been consistently described in IBD. These bacteria contribute to the production of short-chain fatty acids, particularly butyrate, which plays an essential role in maintaining intestinal epithelial integrity and mucosal homeostasis. Additionally, SCFAs have important immunomodulatory effects, including promoting regulatory T-cell development, modulating cytokine production, and suppressing intestinal inflammation. Furthermore, *Bifidobacterium longum*, which has an immunomodulatory role, is also reduced [7,8,9].

Meanwhile, dysbiosis in IBD is associated with an increase in bacteria with pro-inflammatory potential, such as Escherichia coli, particularly adherent-invasive and enterotoxigenic strains (ETEC), *Ruminococcus gnavus*, *Bilophila wadsworthia*, and other bacteria belonging to the *Enterobacteriaceae* family. These microorganisms promote activation of the intestinal immune response through the production of lipopolysaccharides (LPS), disruption of the mucosal layer, and stimulation of pro-inflammatory cytokine production. Additionally, enterotoxigenic strains of *E. coli* can increase epithelial permeability through activation of the NF-κB pathway [10,11,12].

For a prolonged period, the main therapeutic classes used in the treatment of patients with inflammatory bowel disease (IBD) have been represented by 5-aminosalicylic acids, corticosteroids, and immunomodulators such as azathioprine. In recent years, biological therapies and targeted therapies have become essential components in IBD management, through the use of agents that selectively target different components of the immune response, with the aim of inducing and maintaining remission. These include TNF-α inhibitors (for example, infliximab), integrin receptor antagonists such as vedolizumab (an α4β7 integrin inhibitor), IL-12/IL-23 inhibitors (for example, ustekinumab), as well as Janus kinase inhibitors, such as tofacitinib [13,14,15].

Some longitudinal studies have suggested an association between gut microbiome composition and response to biological therapy. Responders to anti-TNF agents, vedolizumab, or ustekinumab have been reported to exhibit greater abundance of beneficial taxa, including *Faecalibacterium prausnitzii*, *Roseburia* spp., and *Eubacterium rectale*, together with greater microbial diversity, whereas non-responders may show increased abundance of potentially pro-inflammatory taxa such as *Escherichia coli* and other *Enterobacteriaceae* [16,17,18,19,20].

Several studies have demonstrated that biological therapy can influence the composition of the intestinal microbiome, leading to increased bacterial diversity and changes in the abundance of certain species associated with intestinal homeostasis [21,22]. Patients in remission under biological treatment exhibited elevated levels of bacteria considered protective, such as *Akkermansia muciniphila*, *Faecalibacterium prausnitzii*, and *Bifidobacterium adolescentis*, which are linked to the integrity of the intestinal barrier, regulation of immune response, and production of short-chain fatty acids [23,24,25]. Conversely, patients with persistent activity or relapse showed a higher abundance of bacteria with potential pro-inflammatory effects, such as *Prevotella* spp. and *Klebsiella pneumonia* [26]. These observations suggest that gut microbiome characteristics may differ according to disease activity and treatment response, although the relationship between biologic therapy and microbiome changes remains incompletely understood.

The study of the gut microbiome during clinical remission is particularly relevant because the absence of overt clinical and biochemical inflammation does not necessarily imply uniformity in microbiome composition. Characterizing microbiome features in patients in remission may therefore help identify microbial patterns associated with clinical and biological control and provide insights into mechanisms potentially underlying incomplete microbiome recovery. A further potential application of microbiome research is the development of strategies for personalized disease management, including biomarker-guided monitoring and targeted modulation of microbial communities [27,28,29].

Given the limited data on gut microbiome characteristics in patients with inflammatory bowel disease who have achieved clinical and biological remission at the time of microbiome assessment during long-term biologic therapy, this study aimed to characterize the taxonomic composition and taxonomically inferred functional microbiome features in this patient population. Particular attention was given to microbial diversity, taxa associated with short-chain fatty acid production, mucin degradation, and lipopolysaccharide production, as well as other microbial groups involved in biologically relevant pathways.

## 2. Materials and Methods

### 2.1. Study Design and Participants

This prospective pilot study included patients diagnosed with inflammatory bowel disease (IBD) who were receiving biological therapy. Participants were recruited from the Department of Gastroenterology of the Emergency Clinical County Hospital of Târgu Mureș, Romania, between October 2025 and March 2026. Eligibility required a confirmed diagnosis of IBD and treatment with biological therapy for at least one year. The study protocol was approved by the Ethics Committee of the Emergency Clinical County Hospital of Târgu Mureș (Approval No. AD 26437, October 2025), and written informed consent was obtained from all participants before enrollment.

### 2.2. Clinical and Laboratory Assessment

To minimize potential microbiome-related confounding, the use of antibiotics or probiotics within the 3 months preceding stool sample collection was a predefined exclusion criterion. Eligibility required a confirmed diagnosis of IBD, treatment with biological therapy for at least one year, and biologic monotherapy without concomitant corticosteroids, immunomodulators, or 5-aminosalicylates.

The Montreal classification was used to classify and evaluate disease phenotypes in patients with Crohn’s disease and ulcerative colitis. For Crohn’s disease, disease location was categorized as L1 (ileal), L2 (colonic), L3 (ileocolonic), or L4 (upper gastrointestinal involvement). For ulcerative colitis, disease extent was categorized as E1 (proctitis), E2 (left-sided colitis), or E3 (extensive colitis).

Disease activity was assessed using clinical and biological inflammatory markers because endoscopic evaluation was not systematically available for all patients at the time of inclusion. Clinical remission was established using disease-specific clinical indices, defined as a Crohn’s Disease Activity Index (CDAI) ≤ 150 for patients with Crohn’s disease and a Mayo score ≤ 3 for patients with ulcerative colitis. Biological remission was supported by inflammatory markers assessed at the time of stool sample collection, including C-reactive protein, erythrocyte sedimentation rate, and fecal calprotectin.

Blood samples from all patients were collected, and a clinical assessment was performed on the same day the stool samples were collected to avoid temporal variability. Inflammatory status was assessed using erythrocyte sedimentation rate (ESR), C-reactive protein (CRP), and fecal calprotectin.

### 2.3. Stool Sample Collection

Fecal samples were collected from all participants according to the laboratory’s standardized collection protocol using stool collection containers containing transport buffer. Care was taken to avoid external contamination during sampling. Samples were transported at room temperature under the conditions recommended by the laboratory until processing.

### 2.4. 16S rRNA Sequencing, Bioinformatic Analysis, and Functional Profiling

Microbiome sequencing was performed using 16S rRNA gene sequencing targeting the V3–V4 region with primers 341F and 805R. Stool samples were collected in a transport buffer and transported at room temperature for molecular processing. PCR amplification and sequencing were performed using an Illumina MiSeq i100 Plus platform. Bioinformatic analysis was performed using QIAGEN CLC Software with the Microbial Genomics Module. Taxonomic profiling was subsequently used to characterize the relative abundance of bacterial taxa.

Bioinformatic processing and taxonomic assignment were carried out using the Qiagen CLC Microbial Genomics Module. The relative abundance of bacterial taxa and the microbiome-derived functional indices reported in this study were generated using the laboratory’s analytical workflow. The reference intervals corresponding to the microbiome parameters reported in the present study were provided by the laboratory and are presented in Supplementary Table S1. These laboratory-specific intervals were used as an interpretative framework and do not represent a matched healthy control population or reference population independently established within the present study.

Functional microbiome profiling included taxonomically inferred bacterial groups associated with butyrate, lactate, and acetate/propionate production, mucin degradation, and lipopolysaccharide (LPS) production. These functional categories were derived from the taxonomic composition according to the laboratory’s analytical workflow and did not represent direct measurements of metabolite concentrations or microbial metabolic activity.

### 2.5. Statistical Analysis

Given the exploratory nature of this pilot study and the small sample size, no formal sample size calculation was performed a priori. Continuous variables were summarized as median (interquartile range, IQR) and range, given the non-normal distribution of most microbiome parameters. Differences between disease subtypes (ulcerative colitis vs. Crohn’s disease) and between biologic agents (adalimumab vs. vedolizumab) were assessed using the Mann–Whitney U test. Associations between functional/taxonomic microbiome markers and patient age or duration of biologic therapy were evaluated using Spearman’s rank correlation coefficient (ρ). Given the exploratory, hypothesis-generating design and the number of comparisons performed, *p*-values were additionally adjusted for multiple testing using the Benjamini–Hochberg false discovery rate (FDR) procedure; both nominal (unadjusted) and FDR-adjusted *p*-values are reported. A two-sided nominal *p*-value < 0.05 was considered indicative of a preliminary signal, while statistical significance was defined as FDR-adjusted *p* < 0.05. Analyses were performed using SPSS (version 26).

## 3. Results

### 3.1. Patient Characteristics

A total of 15 patients with inflammatory bowel disease receiving long-term biologic therapy were included in the study. The cohort comprised eight patients (53.3%) with ulcerative colitis and seven patients (46.7%) with Crohn’s disease. The median age was 49.5 years (IQR, 42–54.5 years). Patients had been receiving biologic therapy for at least one year, with adalimumab administered in eight patients (53.3%) and vedolizumab in seven patients (46.7%). Disease duration was 7 years (IQR, 4–7.5) in patients with Crohn’s disease and 5 years (IQR, 4–6) in those with ulcerative colitis, with a median duration of 5 years (IQR, 4–7) for the overall cohort. The median duration of biologic therapy was 27 months (IQR 21–33), ranging from 14 to 41 months. Inflammatory markers were low across the cohort, with a median ESR of 5.0 mm/h (IQR, 2.5–7.5), CRP of 2.1 mg/L (IQR, 1.0–3.2), and fecal calprotectin of 36.0 µg/g (IQR, 18.2–68.5). All participants had fecal calprotectin levels below 100 µg/g. At the time of stool sampling, all participants were in clinical and biological remission. Demographic and clinical characteristics are summarized in Table 1.

**Figure 1:**
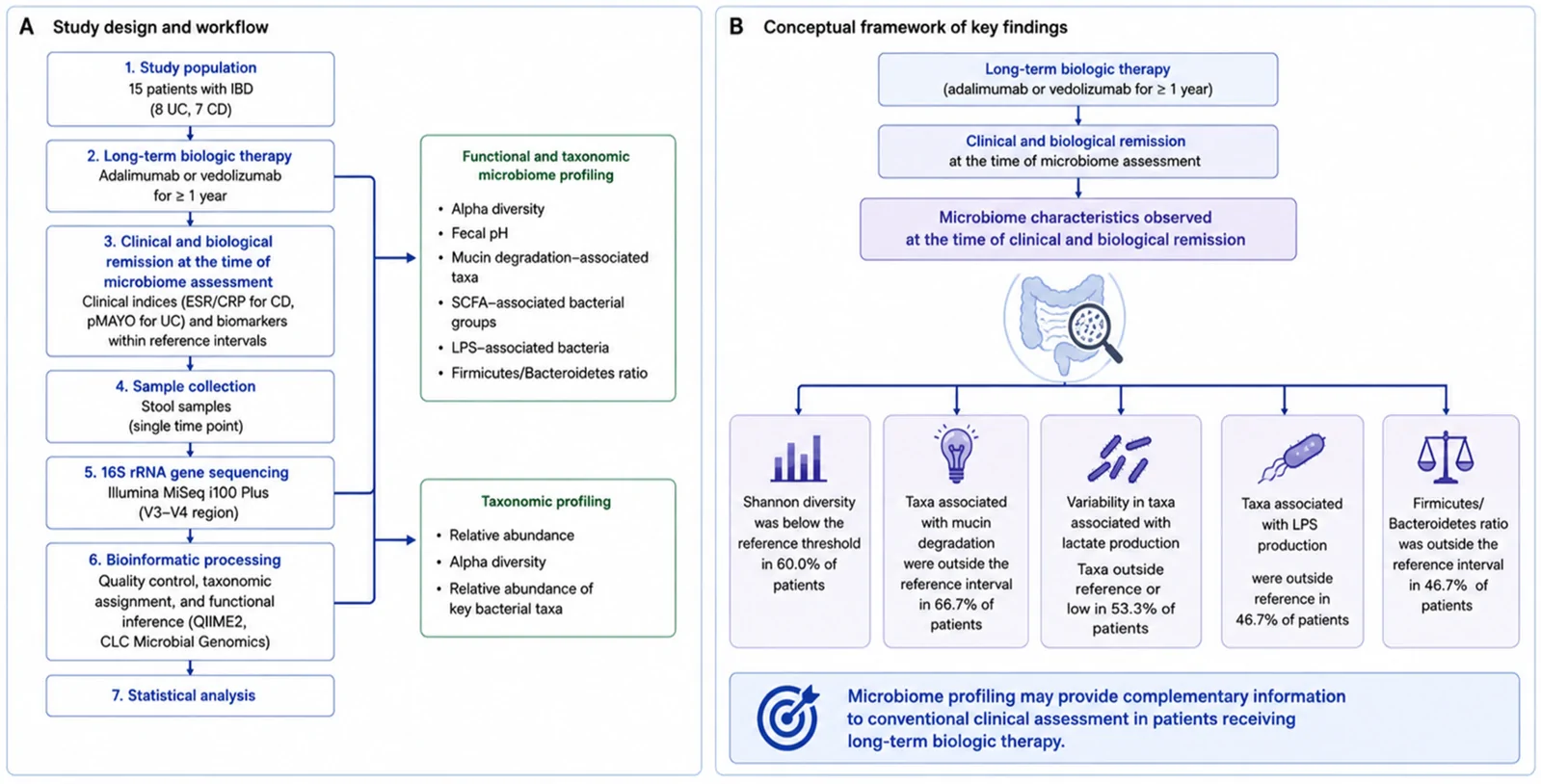
Study design and conceptual framework of the principal findings. (**A**) Overview of patient selection, sample collection, microbiome sequencing, bioinformatic processing, and statistical analysis. Functional and taxonomic microbiome profiling were performed in patients with inflammatory bowel disease receiving long-term biologic therapy. (**B**) Summary of the main study findings, illustrating the microbiome characteristics observed in patients with clinical and biological remission at the time of microbiome assessment and illustrating the potential complementary information provided by microbiome profiling in this research setting.

An overview of the study workflow and the conceptual framework of the microbiome analyses is presented in Figure 1.

### 3.2. Global Functional Gut Microbiome Profile

The global functional gut microbiome profile of the study cohort is summarized in Table 2. Functional microbiome assessment included fecal pH, microbial diversity (Shannon index), Firmicutes/Bacteroidetes ratio, and the relative abundance of bacterial groups involved in butyrate, lactate, and acetate/propionate production, mucin degradation, and lipopolysaccharide (LPS) production.

The median fecal pH was 6.5 (IQR 6.0–6.5), while the median Shannon diversity index was 4.56 (IQR 4.39–4.86). The median *Firmicutes*/*Bacteroidetes* ratio was 3.10 (IQR 2.35–3.20). Regarding taxonomically inferred functional groups, the median relative abundances were 16.9% (IQR 12.25–17.55) for taxa associated with butyrate production, 5.0% (IQR 2.55–13.90) for taxa associated with lactate production, 16.9% (IQR 12.40–17.95) for taxa associated with acetate/propionate production, 5.9% (IQR 1.55–12.35) for taxa associated with mucin degradation, and 1.511% (IQR 0.611–3.930) for taxa associated with LPS production.

Considerable variability was observed across the evaluated microbiome features. Among the parameters with values outside the laboratory-specific reference intervals, the broadest ranges were identified for taxa associated with lactate production (1.2–36.9%), taxa associated with mucin degradation (0.0–18.0%), and taxa associated with LPS production (0.041–7.757%).

### 3.3. Functional Microbiome Characteristics According to Laboratory-Specific Reference Intervals

To descriptively characterize the microbiome profile of the study cohort, each microbiome parameter was classified as either within or outside the laboratory-specific reference interval provided by the laboratory (Table 3). Laboratory-specific reference intervals used for interpretation are provided in Supplementary Table S1.

Fecal pH was within the reference interval in 12 of the 15 patients (80.0%), while taxa associated with butyrate production and taxa associated with acetate/propionate production were within the expected range in 10 (66.7%) and 9 (60.0%) patients, respectively. In contrast, microbial diversity, assessed by the Shannon diversity index, remained below the reference threshold in 9 patients (60.0%). Taxa associated with mucin degradation were outside the laboratory-specific reference interval in 10 patients (66.7%). Taxa associated with lactate production and taxa associated with LPS production were outside the reference interval in 8 (53.3%) and 7 (46.7%) patients, respectively. The *Firmicutes*/*Bacteroidetes* ratio was outside the reference interval in 7 patients (46.7%).

Overall, microbiome features outside the laboratory-specific reference intervals were common despite clinical and biological remission at the time of microbiome assessment. Given the cross-sectional design and the absence of pre-treatment or healthy control samples, these findings describe the microbiome profile observed at the time of sampling and cannot establish the extent of microbiome restoration or the effects of biologic therapy.

### 3.4. Taxonomic Composition of the Gut Microbiome

The relative abundance of the major bacterial phyla is presented in Table 4. The gut microbiota of patients with inflammatory bowel disease receiving long-term biologic therapy was predominantly composed of *Firmicutes* and *Bacteroidetes*, with median relative abundances of 61.51% (IQR 58.23–63.38) and 20.61% (IQR 18.80–23.92), respectively. *Actinobacteria* and *Proteobacteria* were detected at lower median abundances, reaching 8.40% (IQR 4.78–16.88) and 3.35% (IQR 2.58–5.06), respectively.

The abundance of *Proteobacteria* showed marked interindividual variability, ranging from 0.32% to 35.01%, whereas *Actinobacteria* ranged from 2.68% to 22.97%. In contrast, *Verrucomicrobia* and *Fusobacteria* were detected at very low abundances in most patients, with median values of 0.00%, although isolated patients exhibited higher relative abundances.

### 3.5. Functional Bacterial Groups

The relative abundance of representative bacterial taxa involved in intestinal barrier maintenance, short-chain fatty acid production, and inflammation-related metabolic pathways is summarized in Table 5.

Among barrier-associated bacteria, *Faecalibacterium prausnitzii* was the predominant taxon, with a median relative abundance of 10.91% (IQR 7.12–12.24). *Akkermansia muciniphila* was absent or present at low levels in most patients (median 0.00%, IQR 0.00–1.24), with values falling within the reference interval (0.001–3.2%) in only 6 of 15 patients overall—4 with ulcerative colitis and 2 with Crohn’s disease. Within the taxa associated with butyrate production, *Roseburia* spp. exhibited the highest median abundance (1.31%, IQR 0.57–1.55), followed by *Eubacterium* spp. (0.77%, IQR 0.25–0.99). *Butyrivibrio crossotus* was not detected in the study cohort.

Taxa associated with lactate production were generally present at low abundances. The median relative abundance was 0.00% (IQR 0.00–0.06) for *Bifidobacterium* spp. and 0.00% (IQR 0.00–0.00) for *Lactobacillus* spp., although individual patients exhibited detectable values.

Among taxa associated with lipopolysaccharide production, *Sutterella* spp. had the highest median abundance (0.93%, IQR 0.28–2.60) and was detected in 13 of 15 patients, whereas *Escherichia* spp., *Enterobacter* spp., and *Klebsiella* spp. were detected at lower abundances. Sulfate-reducing bacteria were represented by *Bilophila wadsworthia* (median 0.13%, IQR 0.04–0.17) and *Desulfovibrio* spp., the latter with a median relative abundance of 0.00% (IQR 0.00–0.13).

### 3.6. Comparative and Correlation Analysis

No statistically significant differences in functional or taxonomic microbiome markers were identified between patients with ulcerative colitis and those with Crohn’s disease, nor between patients receiving adalimumab versus vedolizumab, after correction for multiple testing (all FDR-adjusted *p* > 0.05). Several nominal differences (unadjusted *p* < 0.05) were observed and are reported as preliminary, hypothesis-generating signals rather than confirmed findings. Taxa associated with mucin degradation (median 12.35% vs. 0.10%, *p* = 0.008), Roseburia spp. (1.55% vs. 0.62%, *p* = 0.018), lactate-producing microbiota (3.35% vs. 17.20%, *p* = 0.040), Eubacterium spp. (0.90% vs. 0.10%, *p* = 0.043), and Bacteroidetes abundance (23.13% vs. 18.50%, *p* = 0.040) differed nominally between ulcerative colitis and Crohn’s disease, respectively. Between biologic agents, nominal differences were observed for Klebsiella spp. (*p* = 0.019), taxa associated with mucin degradation (*p* = 0.036), and taxa associated with lactate production (*p* = 0.040) when comparing adalimumab and vedolizumab, respectively. Age correlated with Desulfovibrio spp. abundance (ρ = 0.53, nominal *p* = 0.040), and duration of biologic therapy showed a non-significant trend toward association with Enterobacter spp. (ρ = 0.47, *p* = 0.077) and Desulfovibrio spp. (ρ = −0.45, *p* = 0.090). None of these associations retained significance after FDR correction.

## 4. Discussion

In this pilot study, we evaluated the gut microbiome profile of patients with inflammatory bowel disease who are on biological treatment, adalimumab and, respectively, vedolizumab. All patients included in the study had received treatment for at least 12 months and were in clinical and biological remission at the time of microbiome assessment.

### 4.1. Gut Microbial Diversity

Gut microbial diversity is closely associated with the integrity and function of the intestinal epithelial barrier. Higher microbial diversity is generally considered a marker of a more stable and resilient gut ecosystem. Previous studies have consistently reported reduced fecal microbial diversity in patients with IBD compared with healthy controls [30,31,32]. Restoration of alpha diversity has been described following successful biological therapy. Sanchis-Artero et al. demonstrated significantly higher Shannon diversity in patients who responded to anti-TNF therapy compared with non-responders, and Fischler et al. also reported improved microbial diversity following effective treatment [32,33].

In our study, gut microbial alpha diversity, assessed using the Shannon diversity index, was within the laboratory-specific reference interval in only 40% of patients. Notably, only two patients with Crohn’s disease had Shannon diversity values within this reference interval. Although all patients were in clinical and biological remission at the time of microbiome assessment, most showed Shannon diversity values below the laboratory-specific reference threshold. Given the cross-sectional design and absence of pre-treatment or matched healthy control samples, this observation should be interpreted as a descriptive characteristic of the cohort rather than evidence of incomplete microbiome restoration.

In this study, we analyzed, in addition to the diversity of the microbiota, a series of parameters that characterize the functionality of the microbiome, such as fecal pH, taxa associated with mucin degradation, taxa associated with lactate production, taxa associated with LPS production, and the *Firmicutes*/*Bacteroidetes* ratio. These additional parameters provide additional taxonomically inferred information regarding microbial groups associated with metabolic pathways, barrier-related processes, and inflammatory potential.

### 4.2. Mucosal Barrier and Mucin Degradation

The intestinal mucus layer constitutes the first line of defense, separating luminal microorganisms from the intestinal epithelium. Mucin-degrading bacteria contribute to normal mucus turnover and help maintain barrier integrity, although changes in their abundance or activity may impair mucosal homeostasis and promote inflammation [34,35].

Taxa associated with mucin degradation were outside the laboratory-specific reference interval in 10 patients (66.7%). This alteration was more frequently observed in patients with UC (75%) than in those with CD (57%), suggesting descriptive differences in mucus-associated microbial characteristics between the two disease phenotypes. However, these differences did not remain statistically significant after FDR correction and should therefore be interpreted as exploratory.

*Akkermansia muciniphila* plays an important role in strengthening the integrity of the mucus barrier and modulating regulatory T cells and cytotoxic T lymphocytes. By contributing to mucus turnover and epithelial homeostasis, this mucin-degrading bacterium helps preserve intestinal barrier integrity. Numerous studies have reported reduced abundance of *Akkermansia muciniphila* in patients with ulcerative colitis, including those in remission; some studies have found no significant differences between Crohn’s disease and ulcerative colitis, and others have reported increased levels in patients with Crohn’s disease [26,36].

Our results showed that depletion of *Akkermansia muciniphila* was more frequent in patients with Crohn’s disease (5/7, 71.4%) than in those with ulcerative colitis (4/8, 50%). Although descriptive differences were observed between the Crohn’s disease and ulcerative colitis subgroups, these findings did not remain statistically significant after FDR correction and should be considered exploratory and hypothesis-generating.

### 4.3. Short-Chain Fatty Acid Metabolism and Taxa Associated with Butyrate Production

Beyond its role in preserving the mucus barrier, the intestinal microbiota also supports epithelial homeostasis through the production of microbial metabolites, particularly short-chain fatty acids.

Lactate is an important metabolic intermediate that is converted into butyrate by bacteria that utilize lactate. Disruption of this process can contribute to impaired production of short-chain fatty acids (SCFA) and intestinal dysbiosis.

Our study found that the taxa associated with lactate production were generally present at low abundances; in 8 (53.3%) cases, they were outside the reference interval. The median relative abundance was 0.00% (IQR 0.00–0.06) for *Bifidobacterium* spp. and 0.00% (IQR 0.00–0.00) for *Lactobacillus* spp., although individual patients exhibited detectable values. However, a recent study of microbiota changes in patients with UC reported elevated levels of lactic acid-producing bacteria [37].

Many commensal Gram-positive bacterial species in the colon can synthesize butyrate from fiber and dietary starch. The two most important groups appear to be *Faecalibacterium prausnitzii* and *Roseburia* spp., which belong to clostridial clusters IV and XIVa, respectively. Butyrate is a short-chain fatty acid (SCFA), and ninety-five percent of the butyrate in the colon is absorbed by colonocytes, for which it serves as a dominant energy source [38].

The three taxa associated with butyrate production evaluated in this cohort, *Faecalibacterium prausnitzii*, *Roseburia* spp., and *Eubacterium* spp., showed descriptive variation according to disease phenotype; however, these observations should be interpreted cautiously given the small subgroup sizes and the lack of statistical significance after FDR correction. In ulcerative colitis, deviations from the reference interval were less frequent and, when present, were exclusively characterized by increased abundance (*F. prausnitzii*: 3/8; *Eubacterium* spp.: 4/8), whereas *Roseburia* spp. remained within the reference range in all patients. In contrast, a predominantly depletion-oriented pattern was descriptively observed among patients with Crohn’s disease, with reduced *Roseburia* spp. in 3/7 patients and depletion of both *F. prausnitzii* and *Eubacterium* spp. in some cases approaching complete absence.

These descriptive observations should not be interpreted as evidence of a disease-specific microbiome pattern. Previous studies have reported reduced abundance of *Faecalibacterium prausnitzii* and *Roseburia* spp. in IBD, particularly in Crohn’s disease [31,37]. However, the present cross-sectional data cannot confirm similar phenotype-specific differences.

The lower abundance of several taxa associated with butyrate production observed in some patients despite clinical and biological remission at the time of microbiome assessment may represent a microbiome characteristic present at the time of sampling. However, in the absence of longitudinal pre-treatment data, it cannot be determined whether these features predated biologic therapy, were modified by treatment, or reflect disease chronicity.

### 4.4. Pro-Inflammatory Microbiota and Microbial Endotoxin Production

Gram-negative bacteria, particularly members of *Proteobacteria*, contribute to intestinal inflammation by producing lipopolysaccharide (LPS), which activates innate immune pathways and leads to epithelial dysfunction. An increase in *Proteobacteria* has consistently been regarded as a hallmark of intestinal dysbiosis in IBD.

In our cohort, *Proteobacteria* abundance was low in most patients (0.32–7.75%). However, one patient with Crohn’s disease had a markedly elevated *Proteobacteria* abundance (35.0%), substantially higher than that observed in the remaining participants. Given the small sample size and the cross-sectional design, this isolated observation should be considered an individual finding and should not be interpreted as evidence of a group-level microbiome pattern or a treatment-related effect.

Regarding LPS-associated taxa, *Sutterella* spp. was the most prevalent taxon in our cohort, detected in 13 of 15 patients (86.7%), though its abundance exceeded the reference threshold (≥1.6%) in only 5 patients (33.3%). Recent evidence suggests that *Sutterella* may act as a pathobiont by inhibiting AhR-dependent IL-22 signaling and impairing mucosal barrier function. Its presence at elevated levels in one-third of patients is a descriptive finding that warrants further investigation in larger cohorts [39]. In contrast, the low but detectable abundances of *Escherichia*, *Enterobacter*, and *Klebsiella* spp. are consistent with previous reports of increased representation of *Enterobacteriaceae* in patients with IBD, even during remission.

In our pilot study, sulfate-reducing bacteria, including *Bilophila wadsworthia* and *Desulfovibrio* spp., were detected at low abundance. These microorganisms produce hydrogen sulfide (H_2_S), which, when in excess, can impair mitochondrial respiration in colonocytes, disrupt epithelial barrier integrity, and promote mucosal inflammation [40,41]. Their presence is notable given the growing evidence linking sulfidogenic bacteria to the pathogenesis of IBD and intestinal barrier dysfunction.

In our cohort, the *Firmicutes*/*Bacteroidetes* ratio was outside the laboratory-specific reference interval in nearly half of the patients, illustrating the heterogeneity of the observed microbiome profiles.

The present findings should also be considered within the broader and evolving field of Microbiota Medicine, which has emerged from the increasing integration of microbiome science with clinical medicine. This interdisciplinary field encompasses the investigation of host–microbiome interactions, microbiome-based biomarkers, microbiota-targeted interventions, and the potential translation of microbiome-derived information into disease prevention, diagnosis, treatment, and individualized patient management [42,43]. In this context, fecal microbiome sequencing represents an important research and biomarker-discovery tool; however, its translation into routine clinical practice remains limited. An international expert consensus has emphasized that evidence supporting the clinical usefulness of microbiome testing remains insufficient for its widespread implementation and has proposed standardized requirements for its potential clinical application [44]. Therefore, while microbiome profiling may provide complementary information for understanding disease-associated microbial patterns and may contribute to future patient stratification, it should currently not be regarded as a standalone diagnostic test or as a validated basis for therapeutic decision-making in IBD.

### 4.5. Limitations

This study has several limitations that should be considered when interpreting the findings. First, the relatively small sample size and the pilot design limited the statistical power to detect subtle differences between Crohn’s disease and ulcerative colitis, as well as between biologic treatment groups. Second, this was a single-center, cross-sectional study, preventing assessment of longitudinal microbiome dynamics and causal relationships between microbial alterations and clinical remission. Third, no healthy control or active IBD comparator group was included, limiting the ability to contextualize the observed microbiome characteristics relative to healthy individuals or active disease. Fourth, microbiome profiling was performed using 16S rRNA gene sequencing, which provides taxonomic characterization but offers limited species-level resolution and does not directly measure microbial metabolic activity or functional gene content. Dietary habits, lifestyle factors, smoking status, body mass index, proton pump inhibitor use, previous intestinal surgery, antibiotic exposure before the exclusion period, and other environmental variables known to influence the gut microbiome were not systematically assessed.

Moreover, endoscopic assessment was not systematically available, and therefore clinical and biological remission in this cohort should not be considered equivalent to endoscopic remission or mucosal healing. This may have influenced the interpretation of the observed microbiome characteristics.

Despite these limitations, the study provides a comprehensive functional and taxonomic characterization of the gut microbiome in a well-defined cohort of patients with inflammatory bowel disease who achieved clinical and biological remission at the time of microbiome assessment after long-term biologic therapy. These findings may contribute to the generation of hypotheses for larger prospective longitudinal studies investigating microbiome dynamics and their relationship with disease activity and treatment over time.

## 5. Conclusions

In this pilot study, patients with inflammatory bowel disease receiving long-term biologic therapy and achieving clinical and biological remission at the time of microbiome assessment exhibited considerable interindividual variability in both taxonomic composition and taxonomically inferred functional microbiome features. Several microbiome parameters were outside the laboratory-specific reference intervals, including microbial diversity and taxa associated with mucin degradation, short-chain fatty acid production, and inflammatory potential.

Given the small sample size, cross-sectional design, absence of pre-treatment samples, and lack of a matched healthy control group, these findings should be interpreted as descriptive and hypothesis-generating rather than as evidence of incomplete microbiome restoration or treatment-related effects. Microbiome profiling may provide complementary information for research into host–microbiome interactions in IBD, but longitudinal studies integrating appropriate control groups and direct functional approaches are required to determine the clinical and biological significance of these observations.

## Figures and Tables

**Table 1:** Demographic and clinical characteristics of patients with inflammatory bowel disease at the time of microbiome assessment.

Characteristic	Overall (n = 15)	UC (n = 8)	CD (n = 7)
Age (years), median (IQR)	49.5 (42–54.5)	45 (40.5–53)	52 (45–54)
Female, n (%)	7	2	5
Male, n (%)	8	6	2
Duration of biologic therapy–months, median (IQR)	27 (21–33)	27 (24–31)	27 (19–35)
Disease duration, median (IQR)	5 (4–7)	5 (4–6)	7 (4–7.5)
Adalimumab, n (%)	8	3	5
Vedolizumab, n (%)	7	5	2
Calprotectin (µg/g), median (IQR)	36 (18.15–68.5)	25.5 (16.23–65.25)	55 (31.5–68.5)
ESR (mm/h), median (IQR)	5 (2.5–7.5)	2.5 (2–5)	8 (5.5–9.5)
CRP (mg/L), median (IQR)	2.1 (1.0–3.2)	1.58 (1.00–2.18)	3.1 (1.65–3.40)
Clinical remission, n (%)	15 (100)	8 (100)	7 (100)
Biological remission, n (%)	15 (100)	8 (100)	7 (100)

**Table 2:** Functional gut microbiome profile of patients with inflammatory bowel disease in clinical and biological remission at the time of microbiome assessment.

Functional Microbiome Marker	Median (IQR)	Range
Fecal pH	6.5 (6.0–6.5)	5.5–8.0
Shannon diversity index	4.56 (4.39–4.86)	3.32–5.28
Firmicutes/Bacteroidetes ratio	3.10 (2.35–3.20)	1.60–4.60
Taxa associated with butyrate production (%)	16.9 (12.25–17.55)	0.0–22.8
Taxa associated with lactate production (%)	5.0 (2.55–13.90)	1.2–36.9
Taxa associated with acetate/propionate production (%)	16.9 (12.40–17.95)	3.6–29.1
Taxa associated with mucin degradation (%)	5.9 (1.55–12.35)	0.0–18.0
Taxa associated with LPS production (%)	1.511 (0.611–3.930)	0.041–7.757

**Table 3:** Functional microbiome markers classified according to laboratory reference intervals.

Marker	Within Range	Outside Range
pH	12 (80.0%)	3 (20.0%)
Shannon diversity	6 (40.0%)	9 (60.0%)
Firmicutes/Bacteroidetes ratio	8 (53.3%)	7 (46.7%)
Taxa associated with butyrate production	10 (66.7%)	5 (33.3%)
Taxa associated with lactate production	7 (46.7%)	8 (53.3%)
Taxa associated with acetate/propionate production	9 (60.0%)	6 (40.0%)
Taxa associated with mucin degradation	5 (33.3%)	10 (66.7%)
Taxa associated with LPS production	8 (53.3%)	7 (46.7%)

**Table 4:** Relative abundance of the major bacterial phyla.

Phylum	Median (IQR), %	Range
*Firmicutes*	61.51 (58.23–63.38)	35.53–75.26
*Bacteroidetes*	20.61 (18.80–23.92)	14.96–29.29
*Proteobacteria*	3.35 (2.58–5.06)	0.32–35.01
*Actinobacteria*	8.40 (4.78–16.88)	2.68–22.97
*Verrucomicrobia*	0.00 (0.00–1.22)	0.00–3.02
*Fusobacteria*	0.00 (0.00–0.00)	0.00–6.23

**Table 5:** Relative abundance of representative bacterial taxa involved in intestinal barrier maintenance, short-chain fatty acid production, and inflammation-related metabolic pathways.

Functional Group	Representative Taxa	Median (IQR), %	Range
Barrier-associated bacteria	*Akkermansia muciniphila*	0.00 (0.00–1.24)	0.00–3.03
	*Faecalibacterium prausnitzii*	10.91 (7.12–12.24)	0.00–15.73
Taxa associated with butyrate production	*Roseburia* spp.	1.31 (0.57–1.55)	0.00–1.63
	*Eubacterium* spp.	0.77 (0.25–0.99)	0.00–1.33
	*Butyrivibrio crossotus*	0.00 (0.00–0.00)	0.00–0.00
Taxa associated with lactate production	*Bifidobacterium* spp.	0.00 (0.00–0.06)	0.00–1.70
	*Lactobacillus* spp.	0.00 (0.00–0.00)	0.00–0.006
Taxa associated with LPS production	*Sutterella* spp.	0.93 (0.28–2.60)	0.00–7.55
	*Escherichia* spp.	0.07 (0.003–0.10)	0.00–2.64
	*Enterobacter* spp.	0.00 (0.00–0.02)	0.00–1.10
	*Klebsiella* spp.	0.00 (0.00–0.01)	0.00–0.05
Sulfate-reducing bacteria	*Bilophila wadsworthia*	0.13 (0.04–0.17)	0.00–0.28
	*Desulfovibrio* spp.	0.00 (0.00–0.13)	0.00–1.03

## Data Availability

The data presented in this study are available on request from the corresponding author.

## Supplementary Materials

The following supporting information can be downloaded at: https://www.mdpi.com/article/10.3390/jcm15187270/s1, Table S1: Laboratory-specific reference intervals for microbiome parameters reported in the present study.
